# Antimicrobial Resistance in *Rhodococcus equi* and the Promise of Synergistic Therapies

**DOI:** 10.3390/antibiotics15030313

**Published:** 2026-03-19

**Authors:** Farzaneh Javadimarand, Pablo Castañera, Blanca Lorente-Torres, Negar Mortazavi, Jesús Llano-Verdeja, Sergio Fernández-Martínez, Helena Á. Ferrero, Luis M. Mateos, Álvaro Mourenza, Michal Letek

**Affiliations:** 1 Departamento de Biología Molecular, Área de Microbiología, Universidad de León, 24071 León, Spain; fjavad00@estudiantes.unileon.es (F.J.); pcase@unileon.es (P.C.); blort@unileon.es (B.L.-T.); jllav@unileon.es (J.L.-V.); sefem@unileon.es (S.F.-M.); halvf@unileon.es (H.Á.F.); lmmatd@unileon.es (L.M.M.); 2 Department of Food Hygiene and Quality Control, Faculty of Veterinary Medicine, Urmia University, Urmia 1177, Iran; n.mortazavi@urmia.ac.ir; 3 Instituto de Biología Molecular, Genómica y Proteómica (INBIOMIC), Universidad de León, 24071 León, Spain; 4 Instituto de Desarrollo Ganadero y Sanidad Animal (INDEGSAL), Universidad de León, 24071 León, Spain

**Keywords:** *Rhodococcus equi*, antimicrobial resistance, multidrug-resistant, synergistic therapies, macrolide-rifampin combination

## Abstract

*Rhodococcus equi* is an opportunistic intracellular pathogen responsible for severe pneumonia in foals and has emerged as an important cause of infection in immunocompromised humans. The treatment of *R. equi* infections in foals relies mainly on the combination of macrolides and rifampin. However, the increasing incidence of multidrug-resistant (MDR) isolates has raised significant therapeutic challenges. The mechanisms underlying this resistance include mutations in target genes, activation of efflux pumps, and biofilm formation, which collectively compromise the efficacy of conventional antibiotics. Recently, growing concern over antibiotic failure has accelerated research into alternative and synergistic strategies to enhance antibacterial efficacy and reduce the development of resistance. Natural and synthetic compounds, as well as optimized antibiotic combinations, have shown promising synergistic effects by enhancing intracellular accumulation, disrupting redox homeostasis, or inhibiting efflux systems. Experimental models employing checkerboard and time-kill assays, as well as redox-sensitive biosensors, have demonstrated that certain antibiotic combinations can influence bacterial susceptibility to antibiotic exposure. Furthermore, integrating molecular tools provides valuable insight into bacterial responses to oxidative and antibiotic stress, paving the way for novel therapeutic designs. This review summarizes the current understanding of the molecular factors contributing to antimicrobial resistance in *R. equi* and assesses new therapeutic approaches aimed at overcoming these challenges. It highlights recent findings on strategies to improve treatment outcomes and manage antimicrobial resistance.

## 1. Introduction

*Rhodococcus equi* is a facultative intracellular bacterium and environmental saprophyte that remains a major pathogen in veterinary medicine, mainly causing pyogranulomatous pneumonia in young foals [1,2,3]. Foals between 1 and 3 months of age are highly susceptible to infection, with infection rates reaching 50–70% in a contaminated environment, where inhalation of aerosolised bacteria from contaminated soil or dust initiates disease [4].

In immunocompromised humans, like those with HIV/AIDS, organ transplant recipients, or individuals on immunosuppressive therapies, *R. equi* can cause opportunistic infections, including cavitary pneumonia, blood infections, and disseminated abscesses, with mortality exceeding 20% in severe cases [5,6]. Its zoonotic potential, supported by genetic similarities between equine and human isolates, is highly relevant to One Health initiatives, linking animal agriculture, environmental persistence, and public health risks [7,8,9].

For the last 40 years, treatment has relied on synergistic dual therapy combining a macrolide (erythromycin, clarithromycin, or azithromycin) with rifampin [10,11,12]. This combination provides effective intracellular penetration, which is essential for eradicating the pathogen from macrophages [13,14,15]. However, the efficacy of these therapies is increasingly compromised by the global rise of antimicrobial resistance, partly driven by the widespread prophylactic and empirical use of antimicrobials in foal management and screening programs [9,10,16]. Additionally, several case series and reviews have reported that human isolates from immunocompromised patients exhibit resistance linked to equine exposure, underscoring the zoonotic significance of antimicrobial-resistant *R. equi* [7,12]. This review synthesises the current evidence on antimicrobial resistance mechanisms, epidemiology, and the evolution of therapeutic approaches to infections.

## 2. The Biology and Pathogenesis of *R. equi*

### 2.1. Taxonomic and Physiological Insights into **R. equi** and Its Environmental Distribution

*R. equi* is a high-G+C Gram-positive, facultatively aerobic coccobacillus of the family Nocardiaceae, closely related to *Mycobacterium* and *Nocardia* [17]. Originally described as *Corynebacterium equi*, it was reclassified into the genus *Rhodococcus* in the 1970s based on 16S rRNA and lipid analyses [18,19]. Recent genomic studies demonstrate the genetic diversity and environmental adaptability of *R. equi*, as well as its placement within a subgenus corresponding to the previously proposed *Prescottella*, allowing the formal citation as *Rhodococcus* (*Prescottella*) *equi* [20,21,22,23]. Virulent equine strains carry plasmid-encoded VapA proteins, distinguishing them from avirulent environmental isolates and serving as molecular markers for diagnostics [17,21]. Morphologically, *R. equi* demonstrates pleomorphism, appearing coccoid in the stationary phase and rod-shaped during growth [17,21]. Its mycolic acid-rich cell wall confers partial acid-fastness and contributes to intracellular persistence within macrophages [24,25]. The bacterium grows efficiently at approximately 30 °C and remains viable across a broad temperature range (10–40 °C), and prefers alkaline conditions (pH 8.5–10) [26,27].

Colonies are smooth, mucoid, and salmon-pink to orange after 48–72 h on nutrient agar [17,18,21], and the bacterium demonstrates catalase and urease positivity, as well as utilisation of short-chain organic acids, inorganic nitrogen, and cholesterol [17,21,28,29]. Its metabolic flexibility, including nitrate/nitrite reduction and hydrogen utilisation, along with resistance to desiccation and oxidative stress, facilitates survival in diverse environments [30]. Ecologically, *R. equi* is ubiquitous in soil, particularly in herbivore manure-rich environments such as equine farms and manure-rich pastures [31,32], and can also be found in the gut and faeces of grazing animals, enabling transmission via ingestion or aerosolisation [33,34]. Warm, dry, and windy conditions favour aerosol spread, contributing to respiratory infections in foals [35]. Horizontal plasmid transfer in soil can convert avirulent strains into virulent forms, highlighting the importance of effective farm management, including dust and manure control, to limit transmission [17,36]. Consequently, effective farm management, including reducing dust and manure accumulation, is essential to control transmission.

### 2.2. Virulence Factors

The pathogenicity of *R. equi*, particularly in foal pneumonia, is primarily determined by host-specific virulence plasmids carrying *vap* genes [21,37,38,39]. The equine plasmid pVAPA (85–90 kb) encodes VapA, a key factor that prevents phagosome-lysosome fusion and mediates intracellular survival in macrophages [40]. Porcine and bovine strains harbour pVAPB and pVAPN plasmids, respectively, encoding VapB and VapN, which mediate host adaptation [41,42,43]. Chromosomal factors also contribute to *R. equi* virulence. The mycolic acid-rich cell envelope and capsule protect against host defences, while the cholesterol oxidase (*choE*) aids nutrient acquisition, and the *mce* loci facilitate cholesterol uptake [24,44]. Biofilm formation and the type VII secretion system enhance persistence and immune evasion [21,45]. Together, plasmid and chromosome-encoded factors enable *R. equi* to thrive as an intracellular pathogen, highlighting the importance of targeted diagnostic and therapeutic strategies in equine veterinary practice.

### 2.3. Epidemiology and Prevalence of **R. equi**

*R. equi* is a soil-borne, facultative intracellular pathogen with a worldwide distribution, reported across Europe, Asia, Australia, and the Americas [46,47,48]. Infection prevalence varies markedly between farms and regions [31,48]. On many farms, the disease is sporadic, but it persists endemically on others. Approximately 10–20% of foals in affected populations develop clinical pneumonia, with a higher incidence reported under conditions that favour bacterial transmission [48]. Subclinical pulmonary lesions may be present in over 50% of foals, though only a proportion progress to overt disease [49]. Mortality is highly variable, ranging from ≤1% with early intervention to 30% during severe outbreaks [31,49].

Surveillance studies highlight the heterogeneous epidemiology. In Türkiye, *R. equi* was detected in 10% of nasal swabs, 22.9% of faecal samples, 29.4% of soil samples, and 5.9% of water samples, with 46.3% of isolates carrying the virulence gene *vapA* [50]. Studies on non-endemic farms provide insight into baseline carriage of *R. equi*. A cross-sectional survey of 1010 healthy horses from 341 non-endemic farms found 1% nasal carriage of *R. equi*, with only one isolate being *vapA*-positive [51]. Comparable studies in India and Ethiopia reported slightly higher carriage (2.6–2.7%) [48]. Most isolates on non-endemic farms are avirulent, reflecting colonisation by environmental strains rather than disease-causing variants [50].

Soil type influences prevalence: Red-Yellow Argisol soils were linked to an eightfold higher prevalence (OR = 8.02), while well-drained soils were associated with lower isolation rates (OR = 0.85) [51]. Foals are particularly important amplifiers of the pathogen, shedding 10^3^–10^8^ CFU/g of faeces, with virulent strains accounting for 10–80% of isolates depending on disease status [31]. Transmission occurs mainly via inhalation of dust containing virulent bacteria, with environmental factors such as dry soil and high horse density increasing risk. Farms act as persistent reservoirs [31,46,49].

Virulence depends on the pVAPA plasmid carrying the *vapA* gene, essential for intracellular survival. At least 14 pVAPA plasmid variants have been identified, showing geographic variation that reflects both local environmental persistence and international horse movement [46]. Across 16 countries, eight main plasmid subtypes have been reported in equine isolates (52 kb, 85 kb types I–V, and 87 kb types I & III), with some subtypes dominant in specific regions [46]. Molecular typing reveals significant genetic heterogeneity among *R. equi* isolates. In the Turkish surveillance study, 29 pulsotypes were identified, grouped into 12 pulsed-field gel electrophoresis clusters (DNA fingerprinting), indicating that infections generally arise from multiple environmental strains rather than a single outbreak clone [50].

Although horses represent the primary host reservoir, *R. equi* has also been reported in a wide range of animal species. A study in Poland detected *R. equi* in 5.1% of wild boars, 0.7% of red deer, and 0.9% of roe deer [52]. In small ruminants such as goats, prevalence appears even lower (approximately 1.1%), although virulent strains have occasionally been identified [53].

Human infections are rare but clinically significant, occurring primarily in immunocompromised individuals, with 80% of cases involving pulmonary infection linked to environmental exposure [5].

### 2.4. Infection Mechanisms and Host Interactions

**Figure 1:**
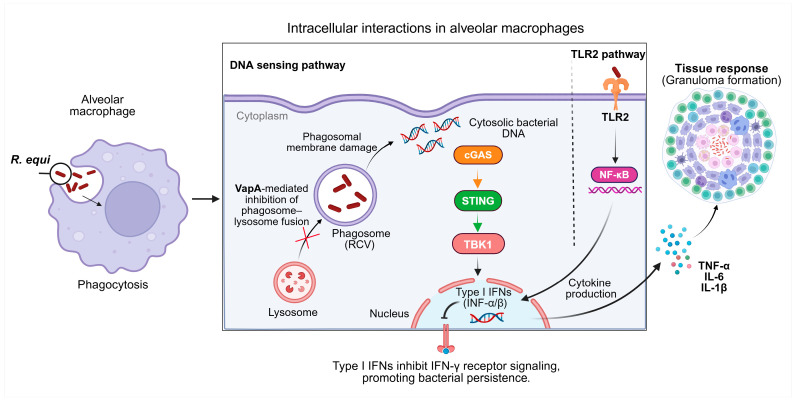
Intracellular interactions of *R. equi* in alveolar macrophages and host immune responses. Following phagocytosis by alveolar macrophages, *R. equi* survives within a specialized phagosome known as the *R. equi*–containing vacuole (RCV). The virulence-associated protein VapA inhibits phagosome–lysosome fusion, allowing bacterial persistence inside macrophages. During infection, damage to the phagosomal membrane can occur, enabling exposure of bacterial DNA to the host cytosol. Cytosolic bacterial DNA activates the cGAS–STING–TBK1 signalling pathway, leading to the induction of type I interferons (IFN-α/β). Type I interferon responses can interfere with IFN-γ receptor signalling, promoting bacterial persistence. In parallel, recognition of bacterial components by TLR2 at the macrophage membrane activates NF-κB signalling and induces the production of pro-inflammatory cytokines such as TNF-α, IL-6, and IL-1β. These cytokines contribute to the development of granulomatous inflammation at the tissue level during pulmonary infection. Created in https://BioRender.com.

Transmission occurs primarily through inhalation of aerosols from contaminated soil or manure [17,32]. Once inhaled, *R. equi* is engulfed by alveolar macrophages, where it forms a specialised *R. equi*-containing vacuole (RCV) that avoids phagosome-lysosome fusion via VapA activity [21,54]. Cytosolic sensing of DNA activates the cGAS-STING-TBK1 pathway (Figure 1), inducing type I interferon (IFN) responses that paradoxically inhibit protective IFN-γ signalling and promote bacterial persistence [1]. Through TLR2-mediated NF-κB activation, immune cells trigger the production of pro-inflammatory cytokines (TNF-α, IL-6, IL-1β), contributing to granulomatous inflammation [55,56]. In foals, disease manifests as chronic pyogranulomatous bronchopneumonia [57]. Foals’ susceptibility has been linked to immaturity of the immune system, including compromised Th1-type responses and delayed or reduced induction of IL-17/Th17 cytokines [58].

Humans are opportunistic hosts, with infections predominantly occurring in immunocompromised individuals. Infection can arise through inhalation, wounds, ingestion, or occupational exposure, with rare cases of human-to-human transmission [5,59]. Pathogenesis in humans resembles that in foals but shows greater variability in virulence factors. Only 20–25% of human isolates express VapA, while VapN-producing strains survive intracellularly, promote IL-4 production, and drive granuloma formation. Prolonged intracellular survival can also lead to malakoplakia due to impaired bacterial clearance [5,60]. Human immune responses mirror those of foals: Th1-mediated IFN-γ is protective, while Th2-mediated IL-4 permits persistence [5]. Pulmonary infection predominates (~80%), with extrapulmonary manifestations and relapse occurring particularly in immunocompromised patients. The detection of animal-associated plasmids highlights the zoonotic nature of *R. equi* [5,59].

## 3. Current Antimicrobial Treatments and Their Limitations

### 3.1. Standard Antibiotic Therapy

**Figure 2:**
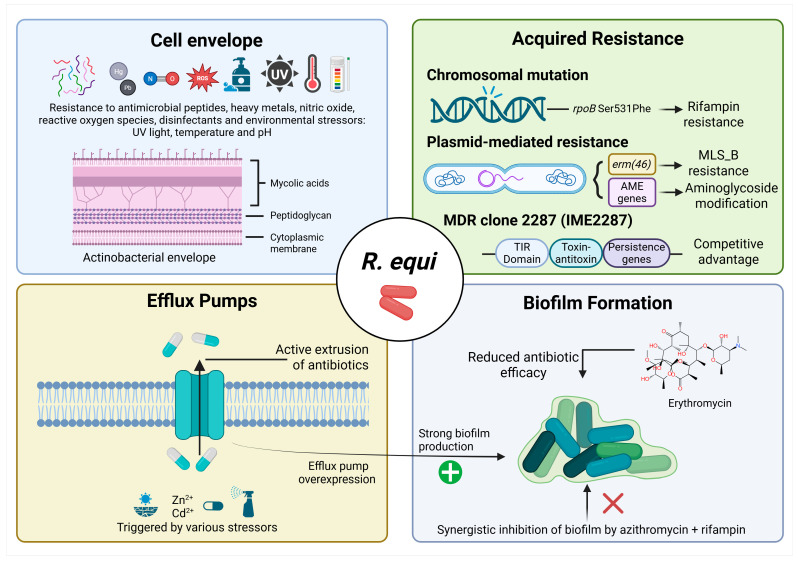
Mechanisms contributing to antimicrobial resistance and persistence in *R. equi*. Multiple intrinsic and acquired mechanisms contribute to antimicrobial tolerance and resistance in *R. equi*. The actinobacterial cell envelope, composed of mycolic acids, peptidoglycan and the cytoplasmic membrane, provides protection against antimicrobial peptides, heavy metals, nitric oxide, reactive oxygen species, disinfectants, and environmental stressors such as UV radiation, temperature, and pH changes. Efflux pumps can actively extrude antibiotics and other toxic compounds, contributing to reduced intracellular drug accumulation and antimicrobial tolerance, and their expression may be induced by environmental stress. Acquired resistance may arise through chromosomal mutations, such as mutations in *rpoB* (e.g., Ser531Phe) conferring rifampin resistance, or through plasmid-mediated mechanisms, including the macrolide resistance gene *erm(46)* and aminoglycoside-modifying enzyme (AME) genes. The multidrug-resistant clone 2287 (IME2287) carries additional genetic determinants, including a TIR-domain protein, a toxin–antitoxin system, and persistence-associated genes that may provide a competitive advantage. In addition, biofilm formation can further reduce antibiotic efficacy by limiting drug penetration and promoting bacterial persistence; combination therapy with azithromycin plus rifampin may synergistically inhibit biofilm formation. Created in https://BioRender.com.

Due to its capacity to survive and replicate within macrophages, *R. equi* requires antibiotics with strong intracellular penetration, a feature that largely determines treatment success. For more than three decades, the standard therapy in both veterinary and human medicine has been a combination of a macrolide, such as erythromycin, clarithromycin, or azithromycin, with rifampin [47,61]. In foals, macrolide-rifampin therapy dramatically improved survival from approximately 20–30% to 60–90% and remains the standard of care [57]. Erythromycin has largely been replaced by clarithromycin and azithromycin, which provide superior oral bioavailability, more favourable pharmacokinetics, and sustained pulmonary and intracellular concentrations [62,63]. Retrospective studies further suggest that clarithromycin–rifampin may be particularly effective, although rifampin co-administration can reduce macrolide levels through metabolic induction [64,65]. Despite its effectiveness, macrolide-rifampin therapy has limitations. Resistance is mainly driven by *erm(46)* and *rpoB* mutations conferring rifampin resistance, including the dissemination of the MDR RE 228 clone among equine populations (Figure 2) [11,66,67,68]. Mass or prophylactic antimicrobial use in subclinical foals promotes MDR emergence, increasing environmental selective pressure. These clones often carry additional resistance genes, limiting treatment options and raising concerns for both animal and public health [12,49,69,70,71]. Adverse effects are common, particularly during prolonged therapy or under high ambient temperatures, including diarrhoea (17–36%), hepatotoxicity (5–10%), colitis, electrolyte disturbances, weight loss, hyperthermia, and respiratory distress [64,72,73,74,75,76,77,78,79].

Pharmacokinetic interactions between rifampin and macrolides may further reduce macrolide exposure, compromising therapeutic efficacy and highlighting unresolved controversies regarding the benefit of combination therapy versus monotherapy [65,66,70,79]. Plasma concentrations of clarithromycin can decrease by up to 40% when co-administered with rifampin in foals [64].

Treatment failure may occur even with susceptible strains due to inadequate drug levels, high bacterial load, host factors, and intracellular persistence. Relapse has been documented in foals after six weeks of therapy, often necessitating treatment up to six months for clearance [9]. The potential antagonism between rifampin and macrolides, and the unclear role of rifampin in preventing macrolide resistance, underscore ongoing controversies and highlight the need for more controlled clinical and pharmacokinetic studies [65,80,81]. Judicious antimicrobial selection, susceptibility testing, and close clinical monitoring are essential to preserve therapy efficacy and guide the development of novel intracellular-targeting agents [47,82,83].

In humans, treatment strategies depend on immune status. Immunocompetent individuals may respond to monotherapy with a macrolide or fluoroquinolone, whereas immunocompromised patients, particularly those with advanced HIV/AIDS, require prolonged dual therapy with intracellularly active agents [48]. Case reports describe successful regimens including clarithromycin–rifabutin, azithromycin–rifampin, or escalation to meropenem when initial therapy fails. Long treatment durations (≥3–6 months) are commonly needed to prevent relapse due to the organism’s intracellular persistence [84]. Macrolide–rifamycin regimens remain most consistently effective, with additional agents (carbapenems, linezolid, vancomycin, fluoroquinolones) reserved for severe or refractory cases [9,61].

### 3.2. Alternative and Combination Therapies for **R. equi**

Given the limitations of standard macrolide–rifampin therapy, other antimicrobial agents with varying intracellular penetration and potential for combination use have been explored as alternative or adjunctive options in the management of *R. equi* infections.

Vancomycin exhibits uniform susceptibility across human isolates, supporting its use in the early management of severe infections [7,85,86]. However, its activity is largely limited to extracellular bacteria due to poor intracellular penetration [83,87].

Carbapenems, particularly imipenem and meropenem, are highly effective against *R. equi* and overcome inducible β-lactam resistance [7,86]. Imipenem is used intravenously for severe human infections, often in combination therapy, whereas meropenem is reserved for refractory cases [7,84,88].

Linezolid demonstrates *in vitro* efficacy against *R. equi*, including macrolide- or rifampin-resistant isolates [3,71,89]. Although linezolid penetrates host cells and achieves effective pulmonary concentrations, its intracellular bactericidal activity against Gram-Positive organisms is limited [90]. Clinical experience in foals remains limited, and human reports describe adverse reactions including marrow suppression and hepatotoxicity [89]. As a priority antimicrobial for human medicine, linezolid should be considered only for life-threatening infections and when susceptibility testing confirms that no effective alternatives are available [71].

Tetracyclines, particularly doxycycline and minocycline, demonstrate potent *in vitro* activity against *R. equi*, including macrolide- and rifampin-resistant isolates. Doxycycline remains effective against foal-derived strains and exhibits synergistic activity with rifampin and macrolides [91,92]. A clinical trial conducted in foals revealed that the combination of azithromycin and doxycycline provides therapeutic efficacy comparable to azithromycin–rifampin therapy [93]. Minocycline shows strong synergy with clarithromycin, retains bactericidal activity against resistant strains, and achieves superior tissue penetration, supporting its potential as an alternative therapy [94,95].

Fluoroquinolones are rarely used as first-line therapy for *R. equi* pneumonia in foals due to the risk of arthropathy. Most clinical isolates remain susceptible to enrofloxacin (<5% resistant) [10], but high-level resistance can emerge rapidly via a single Asp87→Gly mutation in GyrA, conferring cross-resistance to ciprofloxacin [96]. High mutant prevention concentrations of enrofloxacin and ciprofloxacin suggest that using a single fluoroquinolone may promote resistance in *R. equi* [10]. Nevertheless, enrofloxacin exhibits excellent intracellular activity, being the most effective of ten antimicrobials tested against *R. equi* in equine monocyte-derived macrophages at clinically achievable plasma concentrations [97].

Aminoglycosides, including amikacin and gentamicin, retain potent *in vitro* activity against *R. equi*, demonstrating consistent efficacy in isolates from both equine and human sources [72,98,99]. However, their clinical effectiveness is limited, as aminoglycosides poorly penetrate macrophages and cannot efficiently target intracellular bacteria [100,101].

Trimethoprim–sulfamethoxazole (TMP-SMX) was historically used to treat *R. equi* pneumonia in foals but is less effective than macrolide–rifampin therapy and has not been considered first-line since the 1980s [102,103]. While some MDR isolates remain susceptible *in vitro* [3,104,105], pRErm46-mediated resistance, carrying *erm(46)* and *sul1*, reduces its efficacy [66,71]. Therefore, TMP-SMX should be regarded as a secondary option, and its clinical use requires caution even when isolates appear susceptible *in vitro* [103,106].

Tigecycline is highly active against multidrug-resistant *R. equi*, including *erm(46)-* and *rpoB*-mutant isolates [71]. Limited clinical experience has shown success in infections resistant to both macrolides and rifampin [107]. Although veterinary use is restricted due to antimicrobial stewardship considerations, tigecycline remains a leading alternative for managing MDR *R. equi* [71,108].

Newer macrolides, such as gamithromycin, show promising activity in foals, whereas tildipirosin demonstrates poor efficacy [91]. Amoxicillin–clavulanate exhibits limited activity against *R. equi*, with high resistance rates in both equine and human isolates [7,71]. This resistance is intrinsic, likely mediated by β-lactamases and altered penicillin-binding proteins, making it unsuitable for empiric therapy [7].

## 4. Mechanisms of Antimicrobial Resistance in *R. equi*

Because of its unique physiological and structural traits, *R. equi* is inherently resistant to certain antibiotics. Its cell wall contains long-chain mycolic acids that form a hydrophobic barrier, limiting the entry of water-soluble antibiotics (Figure 2). This structure, similar to that of mycobacterial walls, reduces susceptibility to β-lactams by restricting antibiotic access to intracellular targets [99,109]. The mycolic acid layer also increases resistance to environmental stressors, including quaternary ammonium disinfectants, enabling survival in soil and field conditions [110]. Furthermore, *R. equi* exhibits intrinsic resistance to chloramphenicol, mediated by mechanisms such as chloramphenicol acetyltransferases encoded by its genome [111].

In addition to innate resistance, *R. equi* has developed acquired resistance to antibiotics through genetic mutations or horizontal gene transfer [10]. Among effective antimicrobial drugs, macrolides (such as erythromycin, azithromycin, and clarithromycin) and rifampin show significant activity against *R. equi* compared to other antibiotics [112]. Additionally, *R. equi* can develop resistance to aminoglycosides, such as gentamicin, through enzymatic modifications encoded by plasmid genes that produce aminoglycoside-modifying enzymes [111]. The rise of MDR strains is mainly due to the acquisition of conjugative plasmids that carry resistance genes [10]. Specifically, the *erm(46)* gene encodes a 23S rRNA methyltransferase, leading to high-level resistance to macrolides, lincosamides, and streptogramin B antibiotics by altering the ribosomal binding site. This gene is located on the conjugative plasmid pRErm46, which allows efficient horizontal transfer among *R. equi* strains [113]. The pRErm46 plasmid is associated with the dominant MDR clone 2287, which has spread widely through equine populations in the United States [67].

Regarding rifampin resistance, point mutations in the *rpoB* gene, especially at codon 531 (for example, Ser531Phe), alter the RNA polymerase β-subunit. This modification prevents rifampin binding, resulting in high-level resistance [114]. These *rpoB* mutations are frequently selected alongside *erm(46)* during dual macrolide-rifampin therapy, contributing to stable MDR traits in clinical isolates. However, these resistance factors can impose fitness costs, such as slower growth rates in nutrient-poor environments and reduced survival in soil in the absence of antimicrobial pressure [115]. In MDR clone 2287, researchers have identified a novel integrative mobilisable element (IME2287) that carries genes potentially linked to virulence and persistence (Figure 2). This includes a Toll/IL-1 receptor (TIR) domain-containing protein and a toxin-antitoxin system, which may confer a competitive advantage over other MDR strains without substantially impacting fitness [67]. Additionally, efflux pumps in *R. equi* are membrane transporters that actively expel antibiotics. These pumps are functional in some isolates and can be induced by environmental or host-associated stressors, including sublethal exposure to biocides or disinfectants, heavy metal contamination, and subinhibitory concentrations of antibiotics (Figure 2). These systems contribute to both intrinsic and acquired resistance, thereby enhancing bacterial tolerance to antibiotics [116,117,118,119].

Another key factor contributing to the resistance of *R. equi* is the ability to form biofilms. Several studies have shown that biofilm formation varies widely among isolates, indicating that this trait depends on both environmental conditions and genetic backgrounds rather than growth rates or plasmid type [45,120]. Moreover, biofilms are linked to decreased sensitivity to macrolides and rifampicin. Azithromycin is more effective than rifampicin in inhibiting and disrupting *R. equi* biofilms, while their combined use showed a statistically significant synergistic effect. However, even high concentrations of these drugs did not fully eliminate pre-formed biofilms, underlining the biofilm’s protective role [121]. Similarly, another study indicated that overexpression of efflux pump systems, especially those in the Major Facilitator Superfamily (MFS), enhances antibiotic extrusion and biofilm production, reinforcing the connection between biofilm formation and multidrug resistance [122]. A summary of these mechanisms is shown in Figure 2.

## 5. Pharmacokinetics and Pharmacodynamics

The efficacy of antibiotics against *R. equi* depends not only on *in vitro* susceptibility but also on their pharmacokinetic and pharmacodynamic properties, particularly the ability to penetrate and persist within host macrophages [61]. Intracellular accumulation is critical for effective eradication of this facultative intracellular pathogen [123]. Table 1 summarises key PK/PD characteristics of antibiotics commonly used against *R. equi*. Drugs achieving high intracellular concentrations are more effective against intracellular bacteria, whereas some agents exhibit strong extracellular bactericidal activity but limited intracellular penetration, restricting their use primarily to systemic infections. Novel therapies may combine intracellular efficacy with activity against multidrug-resistant strains, although their clinical use is often limited by potential toxicity.

This integrated PK/PD perspective provides a framework for selecting antibiotics based on their distribution, intracellular activity, and synergy potential, ultimately informing optimised therapeutic regimens for veterinary and human *R. equi* infections.

## 6. Emerging Strategies to Overcome Resistance

### 6.1. Natural Compounds Enhance Antibiotic Efficacy Through Multiple Mechanisms, Including Redox Modulation

The rise of antimicrobial resistance (AMR) has intensified the search for novel strategies to enhance the efficacy of existing antibiotics. Natural compounds (NCs), particularly plant-derived molecules such as flavonoids, terpenoids, phenolics, and alkaloids, have emerged as promising adjuncts in combination therapies [133,134]. Although these compounds often exhibit modest antibacterial activity alone, they can significantly potentiate antibiotics by targeting multiple bacterial pathways, including efflux pumps, membrane integrity, quorum sensing, biofilm formation, and redox balance [135,136]. These mechanisms improve antibiotic uptake, enhance bactericidal activity, reduce required doses, delay resistance development, and minimise cytotoxicity, representing a sustainable approach in antimicrobial therapy [137].

When evaluated systematically, NC-mediated antibiotic potentiation falls into three mechanistically distinct classes that differ markedly in their spectrum, intracellular efficacy, evidence strength, and translational limitations. Membrane-disrupting phenolics and terpenoids primarily increase outer-membrane permeability and inhibit biofilm formation, thereby facilitating antibiotic penetration [138,139]. Lipophilic methanol or dichloromethane extracts enriched in these compounds exhibit superior antimycobacterial activity compared with aqueous extracts, an effect attributed to their ability to traverse the lipid-rich mycolic-acid cell wall of actinobacteria such as *Mycobacterium tuberculosis* [140]. However, this class demonstrates robust extracellular synergy but frequently fails to eradicate intracellular bacteria and carries a higher risk of cytotoxicity at effective concentrations.

Redox-active alkaloids and related molecules directly interfere with bacterial antioxidant systems and efflux pumps, inducing a rapid collapse of redox homeostasis and thereby potentiating oxidative killing [137,138,141]. The antimicrobial effects of extracts containing alkaloids, flavonoids, phenolics, and terpenoids often arise from the synergistic combination of direct bactericidal activity and indirect modulation of oxidative stress pathways [142]. This redox-targeting mechanism appears particularly advantageous against intracellular pathogens, where conventional antibiotics frequently exhibit reduced efficacy.

A third class comprises host-directed polyphenolic immunomodulators such as curcumin, which enhance macrophage-mediated clearance of intracellular pathogens [143,144]. Curcumin promotes autophagy and apoptosis, inhibits NF-κB and p38 MAPK signalling, and potentiates macrophage killing of *M. tuberculosis*, while other polyphenols such as bergenin, luteolin, isoliquiritigenin, and baicalein modulate MAPK/JNK pathways, Th1/Th17 responses, macrophage polarisation, NK/NKT activation, and inflammasome suppression, collectively enhancing host immunity [145]. While this approach yields strong *in vivo* efficacy and additional anti-inflammatory benefits, its activity is inherently host-dependent and may be compromised in immunocompromised individuals. 

Evidence from *M. tuberculosis* provides further translational insight into the potential of plant-derived compounds as adjuncts in anti-mycobacterial therapy. Phytochemicals such as curcumin, berberine, betulinic acid, pyridomycin, and various alkaloids exhibit significant antimycobacterial activity through diverse mechanisms, including inhibition of cell wall biosynthesis, disruption of nucleic acid metabolism, modulation of host immune responses, biofilm inhibition, and modulation of host immune responses [146]. Curcumin, in particular, has been extensively characterised for its ability to inhibit *M. tuberculosis* growth, enhance macrophage killing via increased reactive oxygen and nitrogen intermediates, suppress excessive pro-inflammatory cytokine production (TNF-α, IL-6, IL-1β), inhibit biofilm formation, and potentiate first-line antitubercular drugs (isoniazid and rifampicin), potentially enabling dose reduction and decreased toxicity [146,147].

This multi-targeted approach is particularly relevant for intracellular pathogens such as *R. equi*, which persist in macrophages through efflux transporters, antioxidant defences, and biofilm-associated protection [12,148]. Recent evidence demonstrates the potential of NC-based combination therapy. For instance, erythromycin combined with cinchonidine acts synergistically against intracellular *R. equi* in macrophages, leading to greater bacterial killing than either compound alone [141,148].

High-throughput screening studies have further demonstrated that redox homeostasis represents a key vulnerability in *R. equi*. In previous work from our group several compounds selectively inhibited a mycoredoxin-deficient mutant, suggesting that disruption of antioxidant defense pathways may sensitize bacteria to antimicrobial stress [141,148]. While compounds such as ginkgolic acid and morusin inhibited bacterial growth *in vitro*, they did not significantly reduce intracellular survival, likely due to limited access to the intracellular bacterial niche. In contrast, the combination of erythromycin (ERY) with cinchonidine (CIN) produced strong synergistic activity both *in vitro* and in macrophages. Checkerboard assays (FICI = 0.47), time-kill experiments, and intracellular infection models consistently demonstrated enhanced bactericidal activity relative to monotherapy [141].

Mechanistically, real-time redox measurements using the Mrx1-roGFP2 biosensor revealed that cinchonidine rapidly induced oxidative stress, whereas sub-inhibitory erythromycin alone had minimal effects on bacterial redox balance. When combined, however, ERY and CIN triggered a rapid oxidative collapse within minutes, overwhelming bacterial antioxidant defences [138,141].

Importantly, these observations align with the critical role of mycoredoxins in *R. equi* survival. The bacterium relies on three mycoredoxins (Mrx1–3) to maintain redox homeostasis and withstand oxidative stress; a triple mrx-null mutant is unable to survive in macrophages, whereas single or double mutants retain viability, reflecting overlapping functions [138]. Mrx proteins differentially mediate resistance to H_2_O_2_, NaClO, and nitric oxide, and roGFP2 biosensors reveal dynamic redox changes during infection [138]. These findings highlight the critical role of mycoredoxins in counteracting reactive oxygen and nitrogen species and suggest that targeting redox homeostasis could enhance the efficacy of combination therapies against intracellular pathogens [138,144].

Further support comes from widely documented synergistic interactions between natural compounds and antibiotics in actinobacterial pathogens closely related to *R. equi*. A study on rapidly growing mycobacteria (RGM), such as *Mycobacterium abscessus*, *Mycobacterium fortuitum*, and *Mycobacterium chelonae*, found that natural compounds, including trans-cinnamaldehyde, carvacrol, gentisaldehyde, and phloroglucinaldehyde, inhibited bacterial growth and biofilm formation, with MICs ranging from 32 to 512 μg/mL [139]. Time-kill assays showed bactericidal activity for gentisaldehyde and phloroglucinol aldehyde, whereas trans-cinnamaldehyde and carvacrol displayed bacteriostatic effects. Moreover, checkerboard and time-kill analyses revealed synergistic interactions with antibiotics such as amikacin, clarithromycin, and linezolid, with some combinations achieving ≥3 log_10_ CFU reductions. Additional compounds, including citral and geraniol, further enhanced antibacterial efficacy [139].

Microbial natural products further expand the arsenal of potent antimicrobial candidates. Cyclic peptides such as rufomycin I, ecumicin, cyclomarin A, and lassomycin, as well as alkaloids including hapalindole A, fischambiguine B, and suadamin A/B, demonstrate remarkable potency (MIC ≤ 10 µg/mL or ≤50 µM) and high selectivity indices (>10), often targeting novel bacterial mechanisms such as ClpC1 ATPase inhibition or mycolic acid biosynthesis disruption. Notably, rufomycin I exhibits superior potency relative to isoniazid (MIC < 0.004 µM versus 0.23 µM), underscoring the therapeutic potential of natural compounds for drug-resistant actinobacteria [149].

Environmental *Rhodococcus* strains also produce narrow-spectrum antimicrobial metabolites, such as the compound from soil isolate MTM3W5.2, which may synergise with conventional antibiotics, representing a naturally evolved strategy to enhance rhodococcosis therapy [139].

Despite their promise, clinical translation of NCs is currently hindered by poor aqueous solubility, low bioavailability, and rapid metabolic clearance. Advanced pharmaceutical technologies, including nanoparticles, liposomes, and phospholipid complexes, are actively being developed to overcome these pharmacokinetic limitations [146,147,149]. Overall, integrating natural compounds into rational combination therapy regimens represents a compelling approach to enhance antimicrobial efficacy, reduce drug doses, shorten therapy duration, and mitigate the emergence of resistance.

### 6.2. Delivery Systems Based on Synthetic Molecules and Nanoparticles

The growing prevalence of antimicrobial resistance in *R. equi*, particularly in foals, has prompted an urgent exploration of nanotechnology-based drug delivery systems to improve intracellular targeting, stability, and efficacy. Nanotechnology-based drug delivery systems offer promising alternatives by enhancing intracellular targeting, improving drug stability, and enabling controlled release. Various nanoparticle platforms, including liposomes, polymeric nanoparticles, macrophage-targeted systems, host-directed nanotherapies, and stimuli-responsive nanoparticles, have been explored to enhance antibiotic accumulation within infected macrophages and improve efficacy against intracellular pathogens [150,151].

Liposome-based nanoparticles have been among the most extensively studied platforms for antibiotic delivery. Liposomes are phospholipid vesicles capable of encapsulating antimicrobial compounds and facilitating their uptake by host cells. Gentamicin encapsulated within DL-dipalmitoyl-sn-Glycero-3-phospholcholine (DPPC) and PEGylated DPPC-PEG liposomes, which achieve rapid uptake by macrophages, with over 90% of cells internalising liposomes within a few hours, while free gentamicin shows minimal internalisation [152]. PEGylation enhances liposome stability and prolongs intracellular retention, enabling sustained delivery. Confocal microscopy studies demonstrate that PEGylated liposomes effectively colocalise with host cells, even under conditions where the bacterium blocks phagolysosomal maturation [101,152,153]. Liposomal gentamicin (LG) significantly reduces intracellular *R. equi* CFU in macrophages and mouse models, surpassing free gentamicin and clarithromycin–rifampin therapy. Its pH-sensitive liposomes facilitate drug release in acidic phagosomes. However, the use of LG in foals is limited by nephrotoxicity, which can be influenced by illness, dehydration, fever, or NSAID use, similar to free gentamicin. Since LG maintains therapeutic intracellular levels for ≥ 48 h, extending dosing intervals or employing targeted delivery, such as nebulization, could improve safety [152,154,155].

Polymeric nanoparticles, especially those based on Poly(lactic-co-glycolic acid) (PLGA), are promising for enhancing intracellular antibiotic delivery. They encapsulate hydrophobic antibiotics, allow sustained release, and protect against degradation. While data on *R. equi* are still limited, studies of related intracellular pathogens indicate improved intracellular retention and reduced toxicity of rifampin and isoniazid [156,157,158]. PLGA formulations have been explored to enhance macrophage uptake and intracellular pharmacokinetics of rifampin and macrolides [159,160,161].

Recent advances provide additional strategies that could be adapted against MDR *R. equi*. For instance, rifapentine-loaded PLGA-PEG nanoparticles functionalized with mannose (RPT-MAN-PLGA-PEG NPs) significantly improved macrophage uptake, enhanced intracellular clearance of *M. tuberculosis*, lowered the minimum inhibitory concentration, and optimised pharmacokinetics without notable toxicity [162]. Similarly, PLGA nanoparticles functionalized with 1,3-β-glucan (Glu-PLGA NPs) accelerated rifampicin uptake in THP-1-derived macrophages by 62-fold compared to the free drug, enhancing intracellular accumulation and rapid pathogen eradication [157]. Pulmonary delivery of rifampicin-loaded PLGA microspheres also demonstrated high intracellular uptake in alveolar macrophages, strong bactericidal activity against intracellular *Mycobacterium bovis* BCG, and excellent biocompatibility, with minimal inflammatory response [163].

PLGA micro- and nanoparticles are promising tools for drug-resistant *M. tuberculosis*, improving bioavailability, sustaining release, and reducing dosing frequency [164]. Mannosylated Solid Lipid Nanoparticles enabled inhaled rifampicin delivery to alveolar macrophages with controlled release, low cytotoxicity and enhanced uptake via mannose residues, despite slightly reduced respirability [165]. Similar strategies could target *R. equi* within macrophages.

Host-directed nanotherapies (HDN) represent an advanced formulation of host-directed therapies (HDT), in which nanocarriers are used to optimise the intracellular delivery and biological activity of immunomodulatory compounds [166], thereby targeting host immune responses rather than bacteria directly. *R. equi* can survive inside macrophages by interfering with phagosomal maturation and resisting oxidative stress. Using nanoparticles to deliver autophagy activators, TLR agonists, or redox modulating agents could enhance bacterial clearance. Studies show *R. equi* extracellular vesicles activate macrophage TLR2 signalling [56]. Moreover, *in vivo* evidence demonstrated that treatment of neonatal foals with a combination of TLR2 and TLR9 agonists (PUL-042) reduced the clinical severity of pneumonia after an intrabronchial challenge [167]. These findings support HDN as a promising strategy for *R. equi*, potentially improving macrophage-mediated clearance while reducing reliance on antibiotics and limiting the development of resistance.

Smart nanoparticles can sense the environment within infected cells and release their drugs only where needed, such as in acidic, oxygen-stressed, or enzyme-rich compartments. For example, pH-sensitive silica nanoparticles can deliver antibiotics directly inside infected macrophages, thereby improving therapeutic efficacy [168]. Similarly, ROS-sensitive nanoparticles can harness the oxidative microenvironment of infected cells. For example, glycopolymeric nanoparticles constructed from a Poly(propylene sulfide)-block-poly(N-methacryloyl glycine) (PPS-b-PMAG) copolymer preferentially release drugs in response to ROS and are taken up efficiently by macrophages [169]. More recently, studies have developed macrophage-targeted, infection-responsive nanoparticles that exploit both ROS and glutathione for controlled antibiotic release, while also boosting intracellular ROS and pro-inflammatory signalling to improve bacterial killing [170]. Finally, dual ROS- and pH-responsive platforms, such as functionalized MXene nanosheets, have been engineered to precisely deliver antibacterial agents in pathological environments with elevated ROS and acidic pH [171].

Combination nanotherapies aim to enhance antimicrobial efficacy by integrating antibiotic-loaded carriers with macrophage-targeted, host-directed, or stimuli-responsive nanoplatforms that act on both bacterial survival and host–pathogen interactions. Examples include liposomal gentamicin against *R. equi*, polymeric systems co-delivering two anti-tubercular drugs, and infection-responsive nanoparticles designed to combine antibiotic delivery with immunomodulatory effects [150,151,166,170].

## 7. Challenges and Future Perspective

Controlling *R. equi* infections is challenged by the overlap of antimicrobial resistance, diagnostic limitations, and host vulnerabilities, particularly in neonatal foals and immunocompromised humans. These challenges not only undermine therapeutic efficacy but also sustain zoonotic transmission and place a significant economic strain on the equine sector [9,75]. Diagnostic delay remains a major limitation in the management of *R. equi* pneumonia in foals. Because the infection often develops quietly, without apparent symptoms, thoracic ultrasonography identifies early lesions but does not detect all of them. This creates a delicate balance: some foals are treated later than ideal, while others receive unnecessary treatment for minor changes that might resolve on their own [172]. Although serum amyloid A and other biomarker-based screening approaches can be helpful for risk assessment, physiological variability in neonatal foals leads to frequent false positives, reducing their value for guiding antimicrobial use [173]. Limitations in diagnosis have led to widespread prophylactic antimicrobial use on farms, contributing to the development of antimicrobial-resistant strains [73].

In humans, cavitary lesions often mimic mycobacterial pathology, leading to delays in appropriate therapy and culture confirmation that can take several days; multidrug-resistant strains may spread before diagnosis is established [7]. AMR surveillance remains highly variable, although MDR strains are well described in some regions, insufficient reporting in other regions limits global understanding [174]. Environmental reservoirs, such as soil and manure, harbour resistant plasmids, including pREm46, allowing resistance to persist beyond the reach of standard biosecurity measures [175]. Adverse effects, including macrolide hyperthermia, rifampin hepatotoxicity, and pharmacokinetic antagonism, in which rifampin reduces macrolide exposure, erode both treatment compliance and therapeutic synergy [64]. Past overuse of antimicrobials has left residues in feed, driving low-level selection and disrupting the microbiome. Recent findings suggest that antimicrobial resistance in farm soil contributes to higher rates of clinical resistance [176].

Addressing these challenges requires innovative approaches that reduce reliance on traditional antimicrobials. Planned metagenomic analysis of soils from equine breeding regions and genomic characterisation of plasmids in related bacteria will clarify the epidemiology of virulent *R. equi* [34,38]. Clinical trials are needed to evaluate doxycycline’s efficacy and safety in this population; emerging evidence supports doxycycline–clarithromycin as a viable rifampin-sparing alternative [95,177].

Host-directed therapies aim to enhance innate and adaptive immunity, offering effectiveness against drug-resistant or dormant bacteria, reducing the development of resistance, and potentially complementing conventional antibiotics [178]. In neonatal foals, PUL-042 administration increased cytokine production by alveolar macrophages and bronchoalveolar lavage cells and reduced both pulmonary lesions and the duration of clinical pneumonia following *R. equi* infection, providing direct *in vivo* evidence of HDT efficacy [179]. Similarly, intracellular pathogens like *M. tuberculosis* evade immune clearance by subverting processes including phagosome maturation and autophagy, highlighting the relevance of host-targeted strategies for intracellular infections [178].

Integrating host-directed therapies and other interventions with AI-driven genomic surveillance and One Health practices, such as reduced antimicrobial prophylaxis and environmental management, could enable earlier detection of *R. equi*, facilitate targeted interventions, and promote sustainable antimicrobial stewardship [180,181,182,183]. Collaborative efforts among veterinarians, researchers, diagnostic laboratories, and farms are essential to translate these innovations into practice and safeguard both animal and human health.

## 8. Conclusions

The escalating antimicrobial resistance in *R. equi* poses a critical threat to both veterinary and human health, making this zoonotic pathogen a persistent challenge for equine hosts and immunocompromised populations. The growing prevalence of multidrug-resistant strains driven primarily by plasmid-encoded determinants such as *erm(46)* on pRErm46 and *rpoB* mutations has diminished the efficacy of the traditional macrolide–rifampin regimen and raised concerns about zoonotic transmission and environmental persistence. Because resistance continues to expand globally, particularly in agricultural areas where antimicrobial prophylaxis is common, coordinated stewardship and evidence-based treatment strategies have become essential.

Despite these challenges, current advances in synergistic and alternative therapies offer promising avenues to restore antibacterial efficacy. Macrolide–rifampin combinations retain value against susceptible isolates; however, the inconsistent synergy and pharmacokinetic antagonism warrant selective, targeted use. Conversely, macrolide–tetracycline regimens, especially clarithromycin combined with doxycycline or minocycline, have demonstrated consistent synergism, reduced mutant prevention concentrations, and meaningful intracellular activity, supporting their potential as rifampin-sparing alternatives. Similarly, novel adjunctive approaches, including natural compound–antibiotic hybrids and redox-modulating therapies, highlight new mechanisms that enhance intracellular drug accumulation and disrupt bacterial stress responses. Nanoparticle-based formulations further expand these possibilities by improving bioavailability and targeted delivery to infected macrophages.

Looking ahead, innovative strategies such as host-directed immunomodulatory therapies offer additional potential to reduce disease burden and limit reliance on conventional antibiotics. However, these advances must be coupled with robust diagnostic strategies, longitudinal surveillance, and integrated One Health policies to mitigate the spread of MDR clones and preserve therapeutic efficacy.

Overall, overcoming AMR in *R. equi* will require a combined approach that strengthens stewardship, refines synergistic drug combinations, and accelerates the development of novel therapeutics. Through interdisciplinary collaboration and continued innovation, substantial improvements in clinical outcomes and reductions in the global impact of AMR are achievable in the coming decade.

## Figures and Tables

**Table 1:** Pharmacokinetic, pharmacodynamic, and pulmonary distribution characteristics of antibiotics used in foals and horses.

Class	Antibiotics	Dose (mg/kg)	PK Parameters	PK/PD Index	Intracellular Penetration	Limitations/Clinical Notes	References
Macrolides	Azithromycin	10 * PO q24h	*t*_½_: 18–25 h C_max_: NR *T_ma_*_x_: 1.8 h *V_d_*: 11–19 L/kg *F*: 40–55%	AUC/MIC	High; BAL cells & macrophages	Low serum; reduced intracellular activity at acidic pH; combination therapy recommended (azithromycin + rifampicin)	[62,124]
	Clarithromycin	7.5/10 * PO q12h	*t*_½_: 4.0 ± 2.1 h *C_max_*: 0.9–0.94 µg/mL *T_ma_*_x_: 1.5 h *V_d_*: 9.9 ± 2.1 L/kg *F*: 57%	AUC/MIC	Pulmonary distribution; lung levels increase with consecutive dosing	Reduced pulmonary distribution with rifampin; P-gp–mediated intestinal interaction; minimal clinical impact	[63,125]
	Erythromycin	25 * PO q6–8h	*t*_½_: 1–2 h *C_max_*: 1.0–2.9 µg/mL *T_max_*: 0.7–1.7 h *V_d_*: 2.3–7.2 L/kg *F*: 8–36%	fT > MIC	Moderate intracellular penetration; poor extracellular distribution	Levels > MIC ~4–5 h; absorption reduced with feeding	[126,127]
Rifamycins	Rifampin	10–20 * PO, once daily	*t*_½_: 6–8 h *C_max_*: 5.5 µg/mL *T_max_*: 2.5–8 h *V_d_*: 0.85 L/kg *F*: 40–60%	AUC/MIC; Cmax/MIC	High ELF and BAL penetration; lipophilic; accumulates in phagocytic cells	Auto-induction; drug interactions; resistance risk; feeding/GI effects; limited tissue data	[64,128]
Tetracyclines	Doxycycline	10 * PO q12h	*t*_½_: 8.5–11.9 h *C_max_*: 2.54 ± 0.27 µg/mL *T_max_*: 3–5.5 h *V_d_*: ≥1 L/kg *F*: 2.7–17%	AUC/MIC	ELF & BAL similar to serum; intracellular activity present, enhanced with macrolides	Limited foal PK/PD; lower intracellular activity vs. macrolides; clinical efficacy unclear	[129]
	Minocycline	4 * † PO q12h	*t*_½_: 8.5–12 h */ 11–12 h † *C_max_*: 2.3 ± 1.3 µg/mL */0.67 ± 0.26 µg/mL † *T_max_*: 1.5 h */1 h † *V_d_*: 1.0–1.3 L/kg */NR † *F*: 35–75%/NR †	AUC/MIC	High; PELF > plasma; BAL cells > plasma; CSF & synovial fluid detectable†	Limited foal data; tissue and intracellular penetration; adult horse effective for non-ocular infections † rescue/combination use	[94,130]
Fluoroquinolones	Enrofloxacin	5–10 * PO or IV q24h	*t*_½_: 17–18 h *C_max_*: 12 µg/mL *T_max_*: 2.2 h *V_d_*: 2.5 L/kg *F*: 42%	AUC24/MIC	Pulmonary interstitial fluid and ELF 24–80% of plasma; good phagocyte penetration	Cartilage toxicity in juveniles; colitis risk; not recommended for routine foal use	[131]
	Ciprofloxacin	5 † IV 20 PO	*t*_½_: 3.6 ± 1.7 h (PO)/5.8 ± 1.6 h (IV) *C_max_*: 0.60 ± 0.36 µg/mL *T_max_*: 1.46 ± 0.66 h *V_d_*: NR *F*: 10.5 ± 2.8%	AUC/MIC	NR	High adverse event incidence; oral and IV administration can cause diarrhoea, colitis, endotoxemia, and laminitis; use not recommended †	[132]

PK/PD indices of antimicrobial agents against *R. equi*. PK, pharmacokinetics; PD, pharmacodynamics; *t*_½_, elimination half-life; *V_d_*, volume of distribution; *F*, oral bioavailability; *T_max_*, time to maximum plasma concentration; *C_max_*, maximum plasma concentration; AUC, area under the concentration–time curve; MIC, minimum inhibitory concentration; ELF, epithelial lining fluid; BAL, bronchoalveolar lavage; PELF, pulmonary epithelial lining fluid; PO, oral; IV, intravenous; q, every; NR, not reported. PK/PD indices indicate the parameter most closely associated with antibacterial efficacy. Data are primarily derived from foal studies unless otherwise stated. Values may vary with age, formulation, disease status, and drug interactions. References are shown in brackets. Notes: * Data obtained in foals; † data obtained in adult horses. Pharmacodynamic indices were established for *R. equi*, except for doxycycline, which also applies to beta-hemolytic streptococci. References are in brackets.

## Data Availability

No new data were created or analysed in this study. Data sharing is not applicable to this article.
